# A target product profile for a rapid diagnostic test to monitor mosquito gene drive presence and frequency

**DOI:** 10.1186/s12936-026-06065-x

**Published:** 2026-07-22

**Authors:** Prateek Verma, Sebald Verkuijl, Calvin K. Yee, Filippo Randazzo, Damaris Matoke-Muhia, Jonathan Kayondo, Nikolai Windbichler, Michael R. Santos, Frédéric Tripet, John M. Marshall

**Affiliations:** 1 Divisions of Biostatistics & Epidemiology, School of Public Health, University of California, Berkeley, CA USA; 2 Department of Life Sciences, Faculty of Natural Sciences, Imperial College London, London, UK; 3 Leverage Science, LLC, Berkeley, CA USA; 4 Centre for Biotechnology Research and Development, Kenya Medical Research Institute, Nairobi, Kenya; 5 Department of Entomology, Uganda Virus Research Institute, Entebbe, Uganda; 6 Foundation for the National Institutes of Health, North Bethesda, Maryland USA; 7 Swiss Tropical and Public Health Institute, Kreuzstrasse 2, Allschwil, Switzerland; 8 University of Basel, Petersplatz 1, Basel, Switzerland

**Keywords:** *Anopheles* mosquitoes, Gene drive, Malaria control, Mosquito monitoring, Pooled samples, Rapid diagnostic test, Target product profile

## Abstract

Malaria remains a major global health challenge, with over 263 million cases and nearly 600,000 deaths reported in 2023, the majority in sub-Saharan Africa. While conventional interventions such as insecticide-treated nets, indoor residual spraying and antimalarial drugs have reduced transmission, progress has stalled due to the limitations of these interventions and the emergence of resistance. Gene drive-modified mosquitoes represent a promising, potentially transformative vector control strategy, capable of spreading malaria-refractory traits or suppressing mosquito populations. Successful field deployment will depend upon monitoring systems to track the presence and frequency of gene drive constructs as they spread and persist. Current molecular surveillance techniques, though effective, are resource-intensive and reliant on laboratory infrastructure and technical competencies. Here, we make the case for a near-universal and low-cost rapid diagnostic test (RDT) designed to detect gene drive mosquitoes in the field, to complement existing surveillance infrastructure. Two use cases are outlined: (i) to detect the presence of the drive construct in a new population, and (ii) to provide an estimate of drive frequency prior to more accurate laboratory-based measurements. We provide a target product profile for the RDT outlining minimally essential and ideal characteristics, including test procedures, sensitivity, specificity, usability by a range of stakeholders in field settings, and compatibility with pooled testing of mosquito samples. An RDT for gene drive construct detection would support community access and participation in monitoring, enhance regulatory oversight, and promote transparency in field trials, thereby facilitating responsible deployment of gene drive-based malaria interventions.

**Supplementary Information:**

The online version contains supplementary material available at https://doi.org/10.1186/s12936-026-06065-x.

## Background

Malaria has caused the deaths of tens of millions of people over the past 50 years, with an estimated 263 million cases including 597 thousand deaths worldwide in 2023 [1]. Exacerbated by poverty and limited economic development, over 95% of recent cases and deaths have occurred in sub-Saharan Africa. Substantial reductions in malaria transmission have been achieved since 2000 with the widespread adoption of interventions including long-lasting insecticide-treated nets (LLINs), indoor residual spraying with insecticides (IRS), and artemisinin combination therapy drugs (ACTs) [2]; however, progress has stalled over the last decade due to limitations of these interventions, emergence of resistance, and financial constraints [1]. Novel malaria control tools are urgently needed, and gene drive-modified mosquitoes are one of the most promising families of new control tools currently in the development pipeline. Gene drive is a phenomenon whereby “a particular heritable element biases inheritance in its favor, resulting in it being found in most progeny, and over successive generations becoming prevalent in the whole population” [3]. While gene drives may be either natural or synthetic, here we focus on the latter. Gene drive developers are currently focusing on “low-threshold” approaches for targeting the primary malaria vectors in Africa. These systems require the release of only small numbers of modified mosquitoes in order for the construct to spread through a population, thus having a high impact on malaria transmission at relatively low production and delivery costs. Antimalarial effects can be achieved through suppressing mosquito population size [4], or modifying mosquitoes to be less capable of transmitting malaria parasites [5], or a combination of the two [6].

Efficient population suppression has been demonstrated in small and large cage experiments by targeting female reproduction in *Anopheles gambiae* [4, 7, 8]. Additionally, antimalarial population modification strategies have been demonstrated with modifications such as single-chain variable-fragment monoclonal antibodies targeting ookinetes and sporozoites in *Anopheles stephensi, An. gambiae* and *Anopheles coluzzii* [5, 9, 10], and midgut-expressed antimicrobial peptides affecting the development of malaria sporozoites in *An. gambiae* [11]. Mathematical modeling suggests that both suppression and modification strategies could greatly reduce malaria transmission in the field [5, 12, 13]; however, the success of these strategies largely depends upon parameters that can only be reliably measured in the field [14]. Field trials are therefore essential prior to widespread use, and will require acceptance from many stakeholders, including affected communities, local authorities, National Malaria Control Programs (NMCPs)/National Malaria Elimination Programs (NMEPs), governments and regulatory agencies.

Monitoring of the first gene drive field trials will be particularly important, given the novelty of the intervention, the degree of interest surrounding it, and the potential for field trials to proceed in an adaptive manner, based on data collected as the intervention proceeds [15]. Interest in the intervention provides a strong motivation to include community members in monitoring activities, ideally facilitated by established entities such as schools and NMCPs. A robust monitoring protocol will also be needed to adapt trial design in response to data collected during the study. This is particularly important given uncertainties in intervention parameters that will only be resolved during or following a release. For instance, mathematical models to support trial design will initially be based on laboratory data pertaining to the gene drive construct, and ecological data pertaining to the target mosquito species; but the fitness of introgressed gene drive mosquitoes can only be reliably observed in the field, and is expected to be highly influential on the optimal release scheme [16]. When designing a monitoring program, careful consideration should be given to data requirements to support trial objectives, as well as to optimal sampling schemes, mosquito capture techniques, and molecular assays to support these [17]. The nature of the gene drive construct is also relevant, as a suppression gene drive may only have a transient presence at a location before locally removing the target species (and itself), if successful. Following a field trial, intermediate to long-term monitoring may be required by regulators, including in areas beyond the release site.

In the event of widespread deployment as a novel malaria control intervention, monitoring activities can be adjusted to more closely match the needs of malaria control programs in terms of scalable logistics and costs. There is a need for novel technologies and approaches to address the complex monitoring needs of gene drive field trials and post-release monitoring. Here, we argue that the process of gaining public and regulatory acceptance for gene drive field testing would greatly benefit from the development of a simple, low-cost, rapid diagnostic test (RDT), usable by specialists and non-specialists, to distinguish gene drive from non-gene drive individuals within the *An. gambiae* s.l. complex, or in the future, for other species for which gene drive is developed. We define an RDT as any field-deployable diagnostic technology with minimal equipment and engineered for point-of-need application that is used for gene drive detection in mosquitoes. Such a tool would help developers’ engagement activities, support regulators and biosafety agencies, and help build trust by empowering local communities to participate in monitoring efforts. The RDTs will be shared with NMCPs, vector-borne disease control units, and other stakeholders and release programs, thereby enhancing transparency and fostering collaboration. RDTs could also be shared with researchers conducting field trials of other malaria control tools in nearby areas. Accuracy of information collected from these tests will be of key importance, and hence ideal partners in RDT use will have an established presence in the surveillance area, and existing mechanisms for entomological collections, quality control and accountability. This will require an operational plan for implementation, including workflows, training, data management, and funding to support these activities.

**Table:** 

Box 1. Current mosquito sampling and genotyping strategies
Depending on location, a monitoring program that samples mosquitoes monthly can collect anywhere from a few mosquitoes per village per week in the dry season to several hundred or thousands per village per week at the peak of the rainy season(s) [18, 19]. Common adult sampling techniques include pyrethroid spray catches (PSCs), indoor aspirations, and/or the use of traps [20]. Common species are usually identified taxonomically, and target species are stored either in 75–80% ethanol or dried with silica gel ahead of molecular analyses used to characterize morphologically identical members of the *An. gambiae* or *An. funestus* species complexes. Once in the laboratory, DNA is extracted from samples using either cheap extractions based on either buffer digestion and ethanol precipitation with differential centrifugation, or a more expensive affinity column-based extraction. The former approach is used for processing large volumes at lower cost using simple PCR with fragment visualization on agarose gels [21, 22], while the latter approach is used when downstream analyses require purer DNA, such as multiplexed or nested PCR, qPCR, and sequencing-based analyses, or when the target DNA is rare compared to mosquito genomic DNA (e.g., mosquito pathogen detection) [23, 24]
Most African institutions leading and contributing to mosquito surveillance programs, and/or participating in the baseline studies informing gene drive mosquito release programs, have existing workflows for individual mosquito sample processing based around simple PCR-based approaches for species diagnostics, and sometimes bloodmeal analyses, whilst *Plasmodium* detection can be performed either by nested PCR, Taqman qPCR, or ELISA. Hence, in the context of gene drive field trial monitoring, assays for the genetic construct should fit into existing workstreams based on PCR and make use of existing equipment that does not require novel or complex supply chains.
For simple genetic construct detection assays, PCR can provide a presence/absence signal if primers are designed to bind to sequences only found on the transgene. Through adding a primer specific to the wild-type sequence, the resulting three-primer multiplexed PCR assays can be used to discriminate between heterozygotes and homozygotes [25]. Care must be taken to design primers that consider local sequence polymorphisms, as mutations in priming regions are the most common issue affecting the specificity of PCR-based approaches used on field-collected samples [22, 26]. When mosquito samples are abundant, or for large-scale monitoring, genetic construct detection assays that interrogate pools of mosquitoes may be cost-effective. However, detecting rare genetically modified mosquitoes in large pools may exceed the sensitivity of PCR, which cannot reliably detect targets below 1:100. For this, other molecular assays such as loop-mediated isothermal amplification (LAMP) may be better suited. Like PCR, LAMP amplifies specific DNA regions, but operates at a constant 60–65 °C temperature, eliminating the need for a thermocycler and enabling faster turnaround, as LAMP products can be detected using fluorescence with intercalating dyes, or colorimetric methods [27, 28]. LAMP requires strict standard operating procedures (SOPs), dedicated instruments, and workspace specifications, as its high sensitivity makes it prone to contamination. While LAMP assays can be multiplexed, it is more challenging than with PCR since each target requires at least four primers [29, 30]
As molecular technologies, such as DNA biosensors and other rapid nucleic acid detection platforms [31, 32], advance and institutions in malaria-endemic countries gain better access to funding and equipment, new cost-effective genotyping options for monitoring genetic constructs may become available; however, high-tech solutions are not always the most appropriate, as they require dedicated facilities, established supply chains and servicing, and trained personnel

## Use cases for a gene drive rapid diagnostic test

**Fig. 1:**
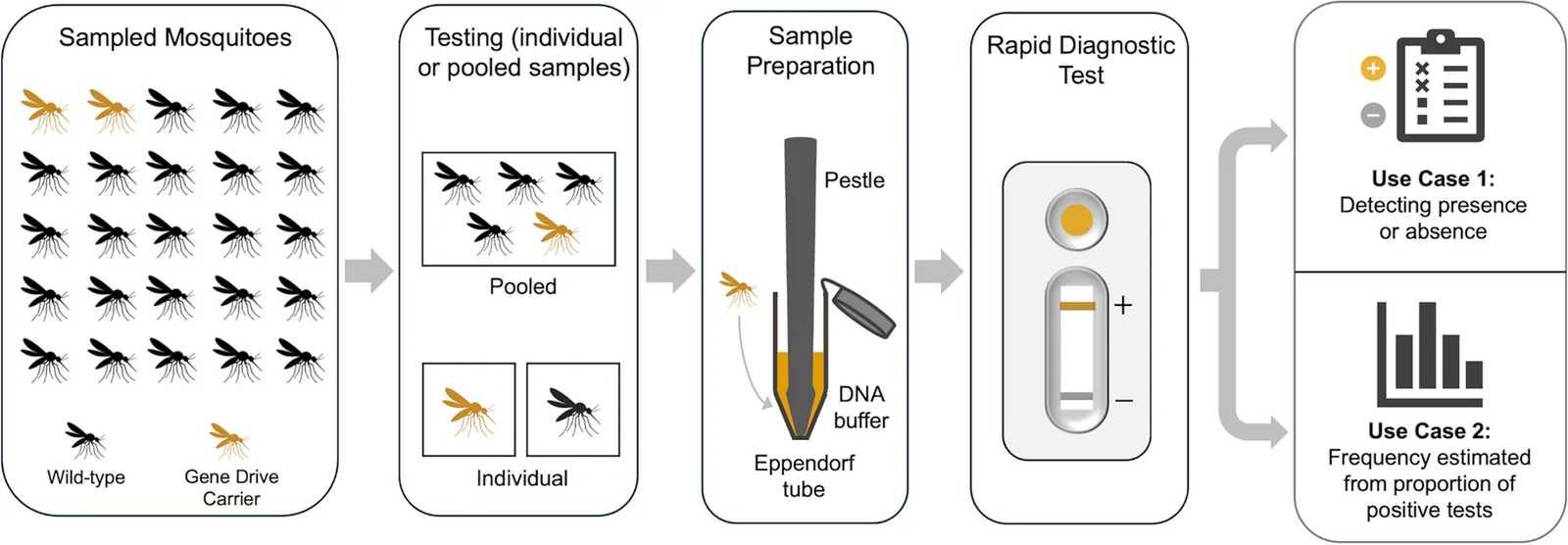
Schematic workflow for performing RDTs on sampled mosquitoes for gene drive monitoring. Mosquitoes can be tested individually or in pools for the two use cases. Use case 1 involves detecting the presence or absence of gene drives, while use case 2 estimates the frequency of gene drive carriers through counts of positive and negative tests

During field releases, gene drive mosquitoes are expected to, over successive generations, disperse outwards from points of release, a process that is influenced by environmental factors such as wind and barriers to movement. Ideally, local mosquito dispersal patterns will be empirically quantified through mark-release-recapture (MRR) studies prior to gene drive release. These pre-release studies can inform the spatial delineation of post-release surveillance zones for monitoring gene drive mosquito presence and frequency [33]. However, mosquito dispersal is frequently characterized as long-tailed, with occasional long-distance movements occurring beyond the predicted range [34], possibly facilitated by factors such as transportation or naturally by wind [35]. In this context, there is a need for a gene drive rapid diagnostic test (RDT)—a tool that can function independently and complementarily to the powerful, but likely less flexible, core field trial monitoring systems (Box 1). We propose two specific RDT use cases illustrated schematically in Fig. 1: (i) detection of the gene drive construct from pools of mosquitoes caught at release and post-release surveillance zones as well as distant areas where the construct is not expected to have reached, and (ii) approximate quantification of a gene drive “carrier frequency” (the proportion of a population either heterozygous or homozygous for the drive) through the use of multiple tests of pooled samples in areas where the gene drive has been confirmed present.

### Use case 1: Detecting the presence of gene drive mosquitoes

This use case focuses on the timely detection of the gene drive construct in a mosquito population at a time when its presence has not yet been confirmed. The RDT would serve as a complement to core monitoring activities of field trials, and as a low-cost tool to enable easy participation of communities in trial and monitoring areas, as well as engagement of specific stakeholder groups, in the context of structured and active engagement activities. Locations where the gene drive has not yet been detected would likely include designated release and post-release monitoring zones, as well as locations beyond the mosquito’s predicted dispersal range. We anticipate the RDT will often be used in locations where gene drive mosquitoes are expected to be absent, and are not part of the core monitoring pipeline or its buffer zones. In this context, RDTs offer distinct advantages, foremost among them the capacity to enable active participation of minimally-trained (simple taxonomic skills) local communities and stakeholders in surveillance activities within defined monitoring areas. Such capacity is essential for confirming designated control areas as true negative controls, ensuring their validity for comparative analyses and for detecting faster-than-expected spread of the gene drive. RDTs provide clear utility in regions lacking access to centralized laboratory facilities or staff trained in molecular assays. Collections would be made through larval habitats, traps placed in houses overnight, or indoor aspiration performed in a house or animal shed. If samples are found to be positive for the gene drive, these should be confirmed through additional RDT tests and/or laboratory-based assays, and these locations may then be included in an existing surveillance pipeline, likely including sample analysis in a centralized laboratory. Last but not least, RDTs could be used in various developers’ stakeholder activities, such as laboratory visits, and various meeting formats including village community engagement. In summary, for use case 1, the RDT would: (i) facilitate detection in low-resource and isolated settings, (ii) enable participation of communities in trial and monitoring areas in the context of structured engagement activities, (iii) provide an initial test for inclusion of a zone as part of the core monitoring system, (iv) provide an approach for detecting the gene drive after the initial trial phase, and (v) be used by developers as a key stakeholder engagement tool.

### Use case 2: Estimating gene drive carrier frequency

This is an extension of use case 1, and takes advantage of the simplicity and flexibility of the RDT to estimate gene drive carrier frequency by performing a limited number of RDTs on mosquito pools of various sizes. This series of RDTs can provide an estimate of gene drive carrier frequency, especially when sampled mosquitoes are representative of the population, and distributed optimally among pools. It is assumed that, when more accurate gene drive carrier or allele frequency estimates are needed, these will involve more extensive and representative sampling, and processing via molecular assays in the laboratory. The RDT-inferred carrier frequency estimates will therefore be provisional, and only seek to provide a rough estimate of gene drive carrier frequency in the population. In this context, for use case 2, the RDT would: (i) facilitate estimation of gene drive carrier frequency in newly-identified positive sites, and (ii) facilitate estimation of gene drive carrier frequency in known positive sites that may not be actively monitored, or would like to be audited by a third party. Collectively, these use cases place certain requirements and restrictions on the design and performance of the test.

## Target product profile for a gene drive rapid diagnostic test

Here, we outline a target product profile (TPP) to guide the design of RDTs for monitoring gene drive mosquitoes, specifying the minimally essential and ideal criteria. We envision use case 1 to be the most common application of the RDT, and hence prioritize its requirements. As we discuss later, we find that the criteria for use case 1 also generally satisfy the criteria for use case 2, and hence, the minimally essential and ideal criteria for both use cases are presented jointly in Tables 1 and 2. Affordability is also an important consideration, and the TPP assumes a per-test cost compatible with large-scale surveillance in resource-limited settings.

**Table 1:** Target product profile for key features of a gene drive rapid diagnostic test

Feature	Minimally essential	Ideal	Considerations
Target user	User with no formal training, provided with *An. gambiae* s.l. samples and RDT instruction leaflet; or user with minimal training in mosquito taxonomy, and RDT instruction leaflet	User with no formal training beyond minimal training in taxonomy, and RDT instruction leaflet	Ideally, the test would be easy-to-use by parties unaffiliated with the release and monitoring efforts that can recognize *An. gambiae* s.l. from other mosquitoes
Target use setting	Dedicated hub near the capture location	Fully on-site, at trap or larval habitat; usable handheld or on knee/ground	The test is intended for use in isolated and impromptu settings
Target species/scope	Validated on primary target species of *An. gambiae* s.l. and other members of the complex, and no false-positives from misapplication	Valid across *An. gambiae* s.l. complex	Preselection of *An. gambiae* s.l. specimens can be taught with minimal training. Assay designed to detect the construct in targeted species of the complex, unless developers or users intentionally target the whole complex
Analyte/target sequence(s)	Any component or product of the gene drive cassette (DNA, RNA, or protein)	Common parts across projects (e.g., Cas9 DNA/mRNA/protein; gRNA scaffold DNA/RNA) with option for construct-specific confirmatory targets	Cas9 and gRNA provide common detection targets for current and likely future implementations. If the analyte is not DNA, higher detection sensitivity will be required, as RNA and proteins will be limited to specific tissues and vary by life stage
Analytical sensitivity	Detect one drive-positive mosquito tested singly; detect one positive in pools of ≥ 10	Same as minimally essential, but with wider pool sizes (e.g., ≥ 25)	Pool size limits would have to be balanced with reduced test cost and increased throughput
Sensitivity	≥ 60% (for mosquitoes tested individually), ≥ 65% (for mosquitoes tested in pools of 10)	≥ 90% (for mosquitoes tested individually), ≥ 92% (for mosquitoes tested in pools of 10)	Minimally essential calculations are based on ≥ 95% probability of detection given a gene drive carrier frequency ≤ 5% and a sample size of 100
Specificity	≥ 99.95% (for mosquitoes tested individually), ≥ 99.5% (for mosquitoes tested in pools of 10)	≥ 99.98% (for mosquitoes tested individually), ≥ 99.75% (for mosquitoes tested in pools of 10)	Minimally essential calculations are based on a sample-wide false positive rate of ≤ 5% and a sample size of 100
Result type & readout	Qualitative (positive/negative), visually readable without instruments	Quantitative/semiquantitative readout; accessible to color-vision–deficient users	Qualitative readout from pooled samples may provide an indication of allele frequency
Sample type	Fresh/damaged/dead adult mosquitoes; larval/pupal acceptable	Same as minimally essential, and including desiccated/frozen specimens	Samples may come from various sources, including traps in which mosquitoes may be dead/damaged/desiccated, or mosquitoes may be stored for some time before analyses
Sample preparation	1.5 mL conical Eppendorf tube with DNA buffer and pestle/dispenser provided	1.5 mL conical Eppendorf tube with DNA buffer and pestle/dispenser provided	
Sample analysis device	Portable	Handheld	RDT can be distributed and shared across sampling areas

**Table 2:** Target product profile for test procedure and additional characteristics of a gene drive rapid diagnostic test

Feature	Minimally essential	Ideal	Considerations
Reagent reconstitution	All reagents supplied, with minimal reconstitution	All reagents are immediately ready to use	Access to pure water or other reagents cannot be relied upon
Time to result from test initiation	< 120 min	< 30 min	Tests are intended to be used on brief visits to field sites
Result validity stability	Up to 30 min	Up to 24 h	Flexibility in readout times will enable parallel workstreams, such as setting up multiple tests simultaneously
Invalid rate	≤ 5% technical failure rate	≤ 1% technical failure rate	Low invalid rate is required due to the relatively high cost of sample collection and brief site visits
**Additional characteristics**			
Operating conditions	15–40ºC, 80% relative humidity	15–40ºC, 80% relative humidity	The test must work in tropical conditions
Test kit stability and storage conditions	No cold chain (15 to 30ºC), test kit stable for at least 6 months	Same as minimally essential, but stable for at least 24 months	The test components should remain stable despite unfavorable transport and storage conditions
Quality control	An internal control to confirm the validity of the test and processing	Same as minimally essential, but with each test having a unique identifier	The competence of the operator will vary, and incorrect use should be indicated; ID will ensure logged results are unique and correctly attributed
Remote connectivity capacity	No additional features	App-based communication to a central database with metadata such as GPS location	Data stored locally and transmitted upon obtaining cell signal. Compiled test results available to relevant stakeholders
Cost per test	Compatible with routine surveillance budgets, indicatively ≤ 5 USD per test for consumables	1–2 USD per test for consumables	Costs exclude personnel, transport, confirmatory laboratory testing, and data management. Monitoring demand scales with the number of sites, sampling frequency, and duration of follow-up

Field compatibility and ease of use are of primary importance for the RDT, as target users ideally include non-specialists such as community members, regulators and other stakeholders, who would require no training beyond reading a manual/leaflet (Table 1). It is envisioned that the test would be performed nearby where mosquito collections take place—i.e., in isolated field locations far from laboratories where more in-depth analyses could take place. In the minimally essential case, the test would detect any component of the gene drive cassette (e.g., Cas9, gRNA, or effector in the case of replacement drives), whether it be DNA, RNA or an expressed protein; but ideally, it would detect sequences that will likely be conserved between different gene drive implementations. For instance, many of the gene drive designs utilized similar DNA sequences that flank the gRNA spacer within the gene drive construct. This approach facilitates a “near-universal” RDT design, mitigating the need for construct-specific iterations. Construct evolution, where all or parts of the gene drive construct may be lost over time due to mutations or recombination events [36], could influence diagnostic performance and thus needs to be considered when designing the RDT. In this context, it would be ideal to target more than one component of the drive construct as this redundancy ensures that detection remains reliable even if the construct undergoes partial deletion, and allows for a more stringent definition of a “true positive.”

For cost-effectiveness and sample processing speed, it is highly desirable that the RDT is able to detect a single positive mosquito in a pool of 10 mosquitoes (or equally, presence of the gene drive at higher positivity rates); but at a minimum, the RDT should be able to detect the gene drive construct when presented with an individual gene drive-modified mosquito. Pooling mosquito samples will likely be necessary to ensure affordability and throughput benchmarks can be met. There should be minimal false positives, as these would both: i) trigger costly follow-up tests, defeating the purpose of the RDT, and ii) risk generating misinformation in community engagement programs. Precise requirements for test sensitivity and specificity in the case of both individual tests and tests of pools of ten mosquitoes at a time are presented in the following section. The RDT should function on a range of mosquito sample types—at a minimum, fresh, damaged or dead adults, larvae or pupae, and ideally, on desiccated and frozen mosquito samples as well. The RDT should be a portable, ideally handheld, device that users can implement with less than half a day of training, and ideally, without training by simply reading a manual/leaflet. Custodianship of devices and considerations regarding interpreting and sharing results should be conveyed to participants prior to RDT use, given the importance of the resulting information to community engagement programs. Interpretation of results should be visual, so that users do not require any additional instrumentation.

Minimally essential and ideal features of the test procedure are presented in Table 2. All reagents for the test should be supplied in a stabilized, ready-to-use format, obviating the need for specialized laboratory infrastructure. The test should ideally yield results within 30 min (at most two hours) as the test is intended to be used on brief visits to field sites, and the validity of the result should remain stable for at least 30 min, but ideally for up to 24 h to allow time for the compiling of multiple test results. The technical failure (non-result) rate for the test should be at most ≤ 5%, and ideally would be ≤ 1%. Non-results increase the required number of tests by a similar amount to the technical failure rate, which increases costs of both the test and sample collection.

Several additional test characteristics are also presented in Table 2. Clearly, the test must be operable in tropical environments where malaria is endemic—i.e., ~ 15–40 °C and ~ 80% relative humidity. There should be no cold chain, and the test kit should be stable for at least six months. Internal controls should confirm that the test is working optimally, and invalid results should clearly be identified. It would be useful, but not essential, for the test to have the capability to connect to a phone with an application capable of sending data to a central database.

Affordability is included as an additional target product characteristic because gene drive monitoring is likely to require repeated sampling across sites and time points. For early field trials, genetic outcomes such as frequency of the construct, spatial spread, and persistence are expected to be central to trial evaluation [17], and monitoring may represent a major cost driver for non-localized gene drive systems [33]. The cost target should also be interpreted relative to centralized molecular testing, as conventional mosquito PCR workflows involving DNA extraction have been estimated to cost approximately 2–4 USD per sample for reagents and consumables alone, depending on the extraction method used [37]. These estimates exclude additional costs related to equipment, sample handling, trained personnel, and laboratory infrastructure, which can be substantial in low-resource settings [38]. Low per-test cost is therefore included in the TPP (Table 2) to support repeated field use, compatibility with pooled testing, and follow-up confirmation by laboratory-based assays when required.

Meeting these performance characteristics in a field-deployable format presents several technical and operational challenges. Achieving very high specificity while maintaining sensitivity across a range of sample types and pool sizes is non-trivial, particularly under field conditions where temperature, humidity, and sample quality may vary. In addition, requirements for minimal training, limited infrastructure, and stable supply chains impose constraints on assay design and implementation. Experience from genetically modified organism detection in agriculture and food systems provides a useful precedent, as screening assays commonly target shared genetic elements, while construct or event-specific molecular assays are used for confirmation and regulatory traceability [39, 40]. Recent CRISPR-based assays for genetically modified crop targets also demonstrate the feasibility of rapid, portable detection of engineered genetic constructs, although such approaches would require validation for mosquito samples and pooled testing [41]. Similarly, *Wolbachia* deployment programs have relied on molecular monitoring to track intervention spread and persistence in mosquito populations, further demonstrating the importance of scalable diagnostic workflows for area-wide vector interventions [42, 43]. A comparable tiered approach is envisaged here, with the RDT providing an initial indication of presence or approximate frequency, followed by more detailed molecular analyses when required.

## Sensitivity and specificity calculations

Here, we consider the minimally essential and ideal test sensitivity and specificity for the two use cases in more detail.

### Test sensitivity for use case 1

In this use case, the RDT would be used to detect the presence of the gene drive cassette in a new population. The false positive rate should be low, as detection of the gene drive will likely trigger costly follow-up collections and analyses. In the minimally essential case, we would like to detect the presence of the gene drive in a population with 95% confidence when the gene drive carrier frequency reaches 5%. Of note, we consider a 5% carrier frequency as a pragmatic design point at which detection is likely even for small sample sizes (e.g., > 20), and at which gene drive spread could still be considered in its early stages. For a gene drive carrier frequency of *x* and a test sensitivity per mosquito of *s*, the probability of detecting the gene drive in a sample of *n* mosquitoes, *p*_*GD*_, is given by:

1 $${p}_{GD}=1-{\left(1-xs\right)}^{n}$$

**Fig. 2:**
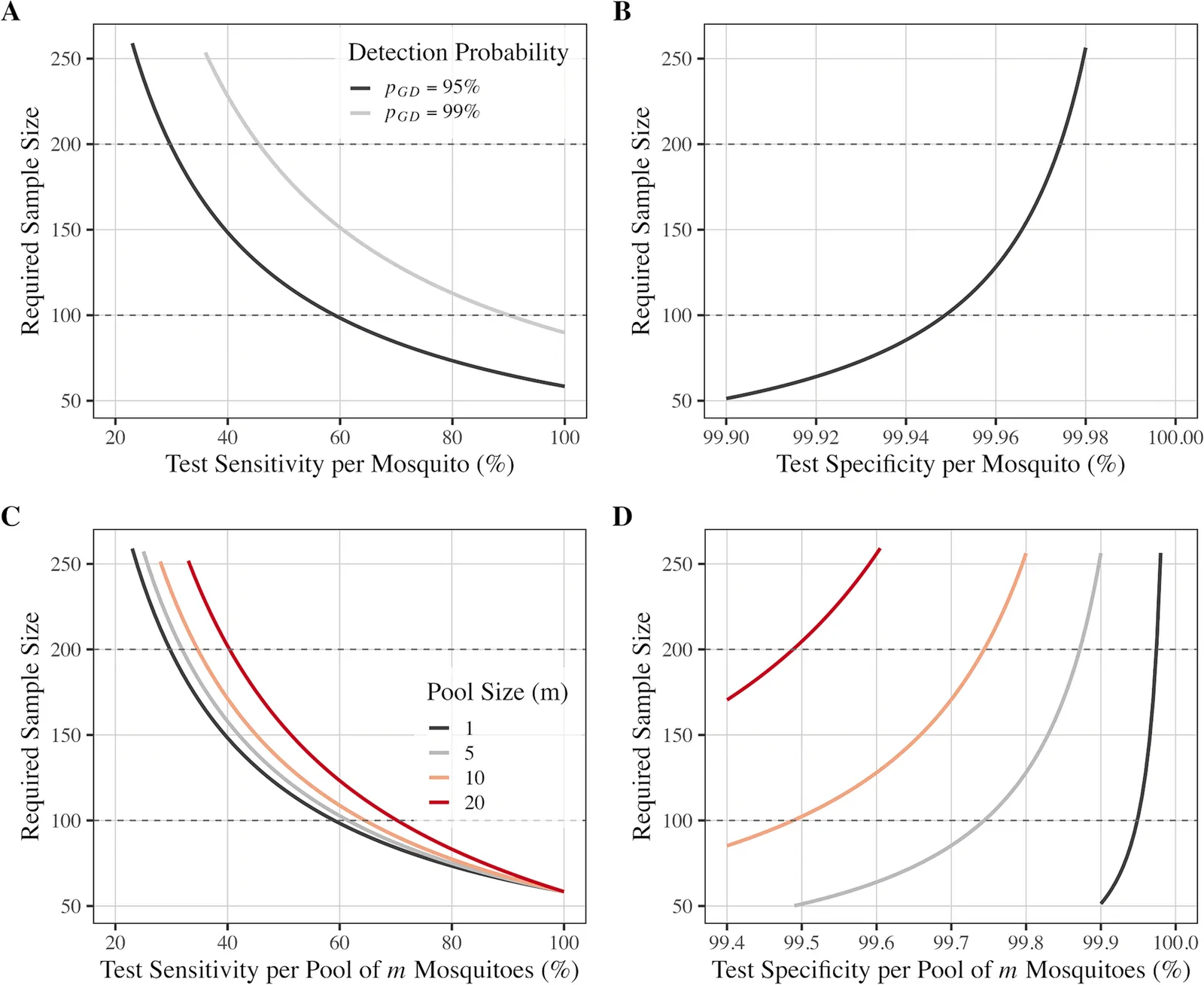
Target sensitivity and specificity parameter values for use case 1. **A** Required sample size as a function of test sensitivity per individual mosquito, assuming we would like to detect the presence of the gene drive in the sample with 95% (minimally essential) or 99% (ideal) confidence when the gene drive carrier frequency reaches 5%. For a sample size of 100, the minimally essential test sensitivity is ≥ 60% and the ideal test sensitivity is ≥ 90%. **B** Required sample size as a function of test specificity per individual mosquito, assuming a sample-wide false positive rate of 5% (Table 1) for a sample size of 100 (minimally essential) or 200 (ideal). **C** Mosquitoes are analyzed in pools of size *m*, and required sample size is depicted as a function of test sensitivity per pool (i.e., the probability of detecting at least one gene drive mosquito in a pool of this size), assuming we would like to detect the presence of the gene drive in the population with 95% confidence (minimally essential) when the gene drive carrier frequency reaches 5%. **D** Required sample size as a function of test specificity per pool of *m* mosquitoes (i.e., one minus the probability of detecting a false positive in a pool of this size), assuming a sample-wide false positive rate of 5% for a sample size of 100 (minimally essential) or 200 (ideal)

For a gene drive carrier frequency of 5% and a minimally essential probability of detection of 95%, Fig. 2A depicts the required sample size as a function of test sensitivity per individual mosquito. Required sample sizes are all in the vicinity of 100. Taking this as a default sample size that is achievable in the field in the rainy season, the minimally essential test sensitivity per individual mosquito is ≥ 60%. We then consider an ideal test sensitivity per individual mosquito of ≥ 90%, which leads to a probability of detection of 99% for a sample of 100 mosquitoes.

At lower gene drive carrier frequencies, larger sample sizes and/or higher test sensitivities are required to achieve comparable detection probabilities, and so the values presented here should be interpreted as illustrative rather than prescriptive. For instance, for a gene drive carrier frequency of 2% and a sample size of 200, a test sensitivity of ≥ 75% is required to achieve a detection probability of 95%. Exploration of alternative scenarios across a range of true carrier frequencies is supported through our open-source R-Shiny application, “TPP Explorer” (available at https://pverma.shinyapps.io/tpp_explorer).

### Test specificity for use case 1

Now let us consider a fully wild-type mosquito population. For a false positive rate per mosquito of *f* (corresponding to a test specificity of one minus *f*), the probability of detecting a false positive in a sample of *n* mosquitoes tested one-by-one, *p*_*FP*_, is given by:

2 $${p}_{FP}=1 - {\left(1-f\right)}^{n}.$$

Let us continue with a total sample size of 100, per the calculations for test sensitivity. Considering a minimally essential sample-wide false positive rate of 5% (Table 1), the minimally essential test specificity per mosquito is ≥ 99.95% (Fig. 2B). Increasing the sample size to 200 mosquitoes, a test specificity of ≥ 99.98% produces a sample-wide false positive rate of 5%. We consider this to be an ideal test specificity per mosquito. Note that, although the minimally essential test specificity of ≥ 99.95% for use case 1 is quite demanding, for a multiplexed diagnostic test, the composite false positive rate, *f*_*MP*_, is equal to the multiplication of false positive rates for its *M* constituent tests, i.e.:

3 $${f}_{MP}={f}_{1} {f}_{2} \dots {f}_{M}.$$

Here, *f*_1_ through *f*_*M*_ refer to the false positive rates of constituent tests 1 through *M*. I.e., for two multiplexed tests of equal specificity, the minimally essential specificity for each test is ≥ 97.8%, and for three multiplexed tests, the minimally essential specificity for each test is ≥ 92.1%.

### Pooled analysis of samples for use case 1

Testing for the presence of a gene drive in a new population will ideally be conducted with pooled samples for speed and cost-efficiency. In this case, the TPP parameters will vary depending upon the number of mosquitoes included in each pool. For a pool size of *m*, the minimally essential and ideal test sensitivity, *s*_*m*_, and test specificity, (1—*f*_*m*_), follow a similar derivation. We first consider the test specificity for a pool of *m* mosquitoes. Given a false positive rate per pool of *m* mosquitoes, *f*_*m*_, the probability of detecting a false positive in a sample of *n* mosquitoes (where *n* is a multiple of *m*) is given by:

4 $${p}_{FP}=1-{\left(1-{f}_{m}\right)}^\frac{n}{m}.$$

Let us consider a total sample size of 100 and a pool size of 10, as an example. Considering a minimally essential sample-wide false positive rate of < 5%, the minimally essential test specificity per pool of 10 mosquitoes is ≥ 99.5%. If we increase the total sample size to 200 mosquitoes, a test specificity per pool of 10 mosquitoes of ≥ 99.75% maintains a sample-wide false positive rate of < 5%. We consider this to be an ideal test specificity for a pool size of 10. Minimally essential and ideal test specificities for a range of pool sizes are depicted in Fig. 2D.

Next, we consider the test sensitivity for a pool of *m* mosquitoes. Again, we would like to detect the presence of the gene drive in the population with 95% confidence when the gene drive carrier frequency, *x*, reaches 5%. The chance that a gene drive carrier is present in a pool of *m* mosquitoes, *x*_*m*_, is given by:

5 $${x}_{m}=1-{\left(1-x\right)}^{m}.$$

We define the test sensitivity for a pool of* m* mosquitoes, *s*_*m*_, as the probability of detecting at least one gene drive mosquito in a pool of this size. The probability of detecting a gene drive mosquito in a sample of *n* mosquitoes (where *n* is a multiple of *m*) is then given by:

6 $${p}_{GD}=1-{\left(1-{x}_{m}{s}_{m}\right)}^\frac{n}{m}.$$

Let us consider the example of a total sample size of 100 and a pool size of 10. For a gene drive carrier frequency of 5%, the probability that a gene drive mosquito is present in a pool of 10 mosquitoes is 40.1%. Considering a minimally essential probability of detection of 95%, the minimally essential test sensitivity per pool of 10 mosquitoes is then ≥ 65%. We consider an ideal test sensitivity per pool of 10 mosquitoes to be ≥ 92%, which would lead to a probability of detection of 99% for a sample of 100 mosquitoes. Minimally essential and ideal test sensitivities for a range of pool sizes are depicted in Fig. 2C. Minimally essential and ideal parameter values for all scenarios explored for use case 1 are summarized in Table 1.

### Test sensitivity and specificity for use case 2

In this use case, the RDT would be used to provide an estimate of gene drive carrier frequency in a population, which could be followed up with more extensive sampling and laboratory processing to provide a more accurate frequency estimate. False positives and false negatives will increase the variance in estimates of gene drive carrier frequency. They will also introduce bias into these estimates; but this can be corrected if the false positive and false negative rates are reasonably well-known in a field setting prior to use. For this use case, we consider two scenarios: (i) measuring gene drive carrier frequency as the drive spreads through a population (e.g., for a carrier frequency of ~ 50%), and ii) characterizing persistence of the drive at high frequency in a population (e.g., for a carrier frequency of ~ 90%). Here, we consider individual tests of mosquitoes; but the case of pooled samples is described in Additional file 1. We are primarily interested in the standard deviation (SD) of our estimate of gene drive carrier frequency, and use a binomial distribution with a known true positive rate, *s*, and a known false positive rate, *f*, to calculate this. For a measured proportion of samples positive for the gene drive, *y*, the estimator for gene drive carrier frequency, $\widehat{p}$, is given by:

7 $$\widehat{p}=\frac{y-f}{s-f}.$$

Considering a sample size, *n*, the SD of the estimator, $\widehat{p}$, is given by:

8 $$SD\left(\widehat{p}\right)=\sqrt{\frac{y\left(1-y\right)}{n{\left(s-f\right)}^{2}}}$$

**Fig. 3:**
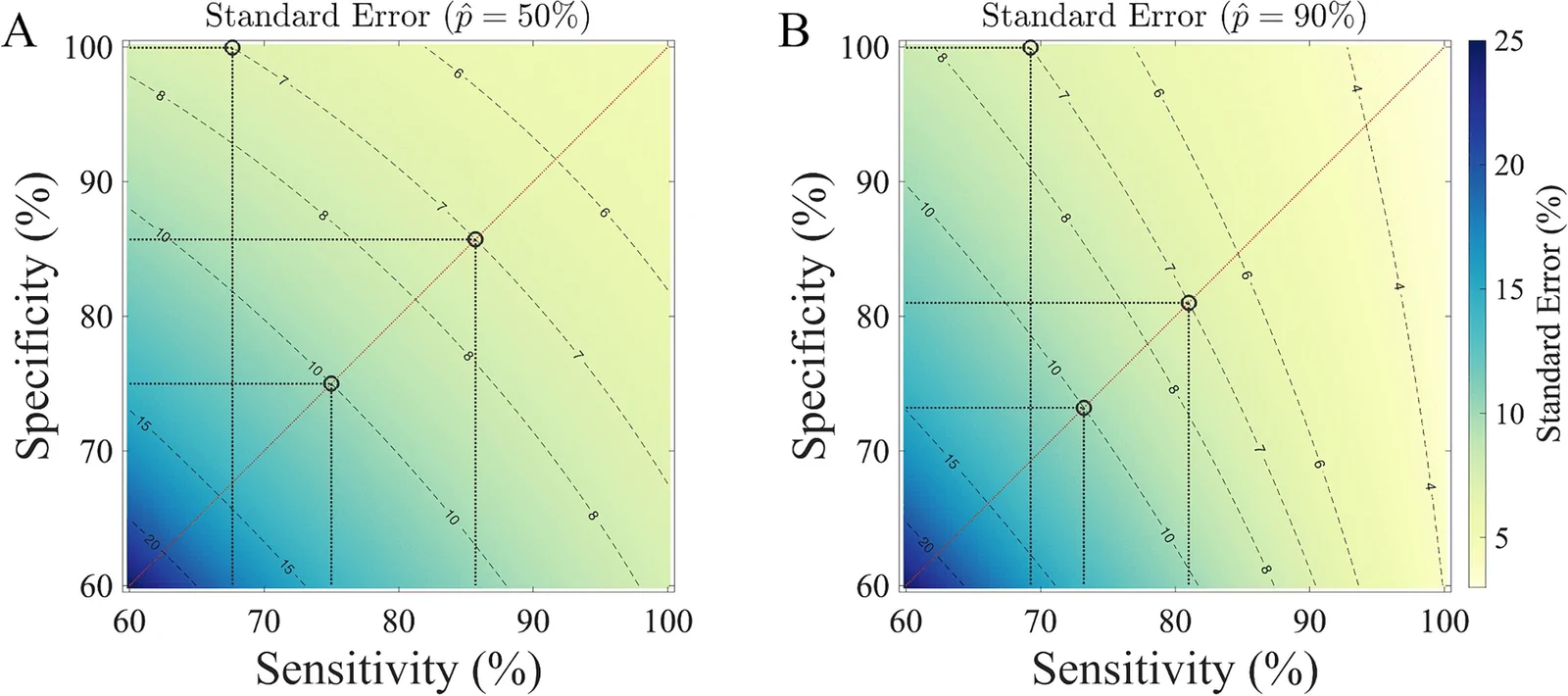
Target sensitivity and specificity parameter values for use case 2. **A** Standard deviation (SD) in estimated gene drive carrier frequency, $\widehat{p}$, as a function of test sensitivity and specificity per mosquito as the gene drive spreads through a population (i.e., for a carrier frequency of ~ 50%), assuming a sample size of 100. Considering test specificity equal to sensitivity, the minimally essential test sensitivity/specificity is ≥ 75% (corresponding to an SD of 10%), and the ideal test sensitivity/specificity is ≥ 86% (corresponding to an SD of 7%). Considering a test specificity of 99.95% (for consistency with use case 1), the ideal test sensitivity is ≥ 68%. **B** SD in estimated gene drive carrier frequency as the gene drive persists in a population (i.e., for a carrier frequency of ~ 90%). Considering test specificity equal to sensitivity, the minimally essential test sensitivity/specificity is ≥ 73% (corresponding to an SD of 10%), and the ideal test sensitivity/specificity is ≥ 81% (corresponding to an SD of 7%). Considering a test specificity of 99.95% (for consistency with use case 1), the ideal test sensitivity is ≥ 69%

Per the previous use case, imagine a sample of 100 mosquitoes, and consider a minimally essential SD of the estimator, $\widehat{p}$, of 10%, and an ideal SD of 7% (of note, the lowest possible SD for this case is 5%, which occurs when the test sensitivity and specificity are both 100%). As the gene drive spreads through the population (i.e., for a carrier frequency of ~ 50%), substituting the minimally essential test sensitivity (60%) and specificity (99.95%) for use case 1 into Eq. 8 yields an SD of $\widehat{p}$ of 7.6%, and substituting the ideal test sensitivity (90%) and specificity (99.98%) for use case 1 into this equation yields an SD of 5.5%. Since these values are less than 10% and 7%, respectively, this implies that the minimally essential and ideal conditions for use case 1 automatically satisfy the conditions for use case 2 as the gene drive spreads through the population. SDs of the estimator, $\widehat{p}$, for a range of test sensitivities and specificities for this scenario are depicted in Fig. 3A. E.g., in the case where sensitivity is equal to specificity, the minimally essential test sensitivity/specificity is ≥ 75% and the ideal test sensitivity/specificity is ≥ 86%, and in the case where test specificity is set to 99.95%, the ideal test sensitivity is ≥ 68%.

Secondly, we are interested in characterizing gene drive carrier frequency as the drive persists at high frequency in a population. In this case, we consider a carrier frequency of ~ 90% and reapply Eq. 8 for the SD of the estimator, $\widehat{p}$. Substituting the minimally essential test sensitivity and specificity for use case 1 into this equation yields an SD of $\widehat{p}$ of 8.3%, and substituting the ideal test sensitivity and specificity for use case 1 yields an SD of 4.4%. Since these values are again less than 10% and 7%, respectively, this implies that the minimally essential and ideal conditions for use case 1 automatically satisfy the conditions for both scenarios for use case 2. SDs of the estimator, $\widehat{p}$, for a range of test sensitivities and specificities for the scenario where the drive persists at high frequency are depicted in Fig. 3B. E.g., in the case where sensitivity is equal to specificity, the minimally essential test sensitivity/specificity is ≥ 73% and the ideal test sensitivity/specificity is ≥ 81%, and in the case where test specificity is set to 99.95%, the ideal test specificity is ≥ 69%.

### Pooled analysis of samples for use case 2

Given the intended use of the RDT to obtain an approximate estimate of gene drive carrier frequency in a field setting where resources are limited, it is likely that pooled rather than individual mosquito tests will commonly be used. Pooling of mosquitoes will increase the SD of the estimator, $\widehat{p}$; but this may be acceptable when the goal is to obtain an estimate of carrier frequency that can be followed up with more extensive and rigorous molecular assays in the laboratory, when needed. For a given sample size and available number of test kits, the optimal distribution of pool sizes depends on gene drive carrier frequency, and the sensitivity and specificity of the RDT for each pool size. A maximum likelihood-based approach to designing optimal pooling strategies for given parameter values and subject to given sample size and test kit constraints is described in Additional file 1. Here, to evaluate each pooling design, mosquito sampling and pooled testing is simulated *n*_*rep*_ times, and carrier frequency is estimated using a maximum likelihood framework applied to the replicate-level pooled test outcomes. The SD of the estimator, $\widehat{p}$, is then derived from the inverse of the observed Fisher information, and pooling designs are ranked in terms of their SD.

We developed an R-Shiny application, “TPP Explorer”, to explore optimal pooling designs to minimize the SD of the estimator, $\widehat{p}$, for a given sample size and available number of RDTs. The optimal distribution of pool sizes is highly dependent on gene drive carrier frequency—at high frequencies, larger pools become saturated with positive tests and hence individual tests or smaller pools are more informative; while at low frequencies, larger pools are informative when paired with pools of different sizes. Consider, for instance, a sample size of 100, 20 test kits, a test sensitivity (independent of pool size) of 90% (the ideal value for use case 1), a test specificity (independent of pool size) of 99.95% (the minimally essential value for use case 1), and possible pool sizes of one, two, five and 10. For a carrier frequency of 10%, the optimal pooling distribution is a single test of 10 pooled mosquitoes, 17 tests of five pooled mosquitoes, and two tests of two pooled mosquitoes, which results in an SD of 3.7%. For a carrier frequency of 90%, the optimal pooling distribution is 20 tests of individual mosquitoes, which only makes use of a fifth of the sampled mosquitoes, but results in an SD of 9.7%.

A trade-off exists between the desired precision of the carrier frequency estimate and the number of test kits used; but 20 test kits are generally sufficient to obtain an SD in the vicinity of 10% for the above-listed parameters (a sample size of 100, sensitivity of 90%, and specificity of 99.95%). E.g., for a carrier frequency of 50%, the optimal pooling with 20 test kits results in an SD of 11.6%, which, if desired, can be reduced to 10.0% by increasing the number of test kits to 27.

These results assume that mosquitoes are sampled randomly from a well-mixed population, and that individuals within each pool represent independent samples from the underlying population. In practice, mosquito populations are spatially structured, and collections are often concentrated at a small number of trapping locations. Under such conditions, detection probability and frequency estimates depend on how sampling locations relate to the underlying population distribution. In spatially heterogeneous populations, achieving a desired detection probability may require larger or repeated samples, or sampling distributed across several locations rather than relying on pools constructed from a small number of collection sites. Where collections are clustered, constructing pools from mosquitoes obtained across multiple traps or households within a site will provide a more representative estimate of population frequency. These considerations do not alter the target sensitivity and specificity values outlined in the TPP; but they do affect how these performance characteristics are achieved in practice. Pooling designs also have direct implications for the number of tests required, and hence overall monitoring costs, which we consider in the next section.

### Cost considerations and monitoring effort

While precise costing is beyond the scope of this study, it is useful to consider the scale of monitoring effort implied by the proposed use cases of RDT. As an illustrative scenario, consider monitoring across 10 sites with sampling of 100 mosquitoes per site, which is consistent with achievable field collections. These estimates represent a simplified scenario of RDT requirements only, rather than the full monitoring effort, which may include additional sampling and confirmatory laboratory testing, with RDT-based testing complementing rather than replacing centralized laboratory analyses. For use case 1, which focuses on detection at low or unknown frequency, pooled testing with pools of 10 mosquitoes implies approximately 10 tests per site per sampling round, corresponding to on the order of 100 tests across all sites. For use case 2, applied during targeted follow-up sampling campaigns after detection, approximately 20 pooled tests per site are sufficient to achieve a standard deviation of about 10% for a sample size of 100 mosquitoes. This corresponds to on the order of 100–200 tests per sampling round across all 10 sites.

Overall, these estimates suggest that even a relatively small trial may require on the order of 10^3^–10^4^ RDTs per year, assuming monthly sampling for use case 1 and more frequent sampling for use case 2. These requirements would scale with sampling intensity and the number of monitored sites. Experience from *Wolbachia* deployment programs suggests that diagnostic and monitoring requirements can become substantial in area-wide vector interventions, with monitoring intensity depending on trial endpoints, spatial scale, and duration of follow-up [42, 43]. At the ideal cost target of USD 1–2 per test specified in Table 2, these testing requirements corresponds to several thousand to tens of thousands of dollars annually, excluding personnel, transport, and confirmatory laboratory analyses. These calculations highlight that even modest per-test costs can translate into substantial programmatic expenses when scaled across space and time, making pooling and low-cost RDTs essential for maintaining feasible monitoring budgets.

## Conclusions

Development of an RDT for detecting gene drive mosquitoes will be a critical step toward engaging communities in monitoring activities, enhancing regulatory oversight, and promoting transparency in field trials. Here, we define a TPP for this monitoring tool, establishing minimally essential and ideal criteria to guide test kit development. We consider two use cases: i) detection of a gene drive construct at a location where its presence has not yet been confirmed, and ii) approximate estimation of gene drive carrier frequency, which could be followed up with more extensive laboratory-based assays, when needed. We find that sensitivity and specificity criteria for use case 1 automatically satisfy criteria for use case 2, and hence present these jointly. Field compatibility and ease of use are of primary importance, and the RDT would ideally detect a molecule (DNA, RNA or protein) that would be conserved between the various gene drive implementations under consideration. Test specificity should be very high (at least ≥ 99.95%), as false positives may create unease, and likely trigger costly follow-up collections and analyses. Test sensitivity should be moderate-to-high (at least ≥ 60%, and ideally ≥ 90%). Accurate functioning for pooled testing is ideal, as this would facilitate the use of fewer test kits in resource-limited settings. An RDT with these characteristics would complement existing molecular surveillance systems, extend monitoring capacity to remote settings, and contribute to building public trust, an essential requirement for the progression of gene drive interventions from laboratory to field. Prior to implementation, thought must go into data reliability and actions that may be triggered by an unexpected positive or negative RDT result. Ultimately, a field-compatible, low-cost RDT would advance safe and ethical implementation of gene drive strategies, and hence contribute to global malaria elimination efforts.

## Supplementary Information

Additional file 1. Optimizing pooled testing for estimation of gene drive carrier frequency

## Data Availability

All codes and source files used to produce the figures in this manuscript are available on GitHub at https://github.com/prateek1verma/tpp-explorer. We have also developed an interactive R-Shiny application called “TPP Explorer,” available at https://pverma.shinyapps.io/tpp_explorer/, which allows users to explore how different test performance characteristics and sampling parameters influence the design and interpretation of diagnostic strategies for gene drive detection. The tool supports flexible investigation of a variety of scenarios, extending beyond the specific examples presented in Figs. 2 and 3, including optimal pooling scenarios to minimize SD in the estimator, $\hat{p}$, for a given sample size and available number of RDTs. It also allows users to visualize estimated gene drive prevalence across a range of true prevalence values for any pooled test design, with customizable sensitivity and specificity for each pool size.

## Abbreviations

ACT: Artemisinin combination therapy drug
DNA: Deoxyribonucleic acid
ELISA: Enzyme-linked immunosorbent assay
gRNA: Guide ribonucleic acid
IRS: Indoor residual spraying with insecticides
LAMP: Loop-mediated isothermal amplification
LLIN: Long-lasting insecticide-treated net
mRNA: Messenger ribonucleic acid
MRR: Mark-release-recapture
NMCP: National malaria control program
PCR: Polymerase chain reaction
PSC: Pyrethroid spray catch
qPCR: Quantitative polymerase chain reaction
RDT: Rapid diagnostic test for the gene drive construct
RNA: Ribonucleic acid
SOP: Standard operating procedure
SD: Standard deviation
TPP: Target product profile

## References

[1] World Health Organisation. World malaria report 2024. Geneva, Switzerland: WHO Press; 2025.

[2] Okello G, Aucamp M. Malaria: a review on its current epidemiological status and management strategies. Malar J. 2026;25:132. 10.1186/s12936-025-05748-1 PMC12958513 41639729

[3] Alphey LS, Crisanti A, Randazzo FF, Akbari OS. Opinion: Standardizing the definition of gene drive. Proc Natl Acad Sci U S A. 2020;117:30864–7. 10.1073/pnas.2020417117 PMC7733814 33208534

[4] Kyrou K, Hammond AM, Galizi R, Kranjc N, Burt A, Beaghton AK, et al. A CRISPR-Cas9 gene drive targeting *doublesex* causes complete population suppression in caged *Anopheles gambiae* mosquitoes. Nat Biotechnol. 2018;36:1062–6. 10.1038/nbt.4245 PMC6871539 30247490

[5] Carballar-Lejarazú R, Dong Y, Pham TB, Tushar T, Corder RM, Mondal A, et al. Dual effector population modification gene-drive strains of the African malaria mosquitoes, *Anopheles gambiae* and *Anopheles coluzzii*. Proc Natl Acad Sci U S A. 2023;120:e2221118120. 10.1073/pnas.2221118120 PMC10629562 37428915

[6] Verkuijl SAN, Del Corsano G, Capriotti P, Yen P-S, Inghilterra MG, Selvaraj P, et al. A suppression-modification gene drive for malaria control targeting the ultra-conserved RNA gene mir-184. Nat Commun. 2025;16:1–14. 10.1038/s41467-025-58954-5 PMC12032250 40280899

[7] Hammond A, Galizi R, Kyrou K, Simoni A, Siniscalchi C, Katsanos D, et al. A CRISPR-Cas9 gene drive system targeting female reproduction in the malaria mosquito vector *Anopheles gambiae*. Nat Biotechnol. 2016;34:78–83. 10.1038/nbt.3439 PMC4913862 26641531

[8] Hammond A, Pollegioni P, Persampieri T, North A, Minuz R, Trusso A, et al. Gene-drive suppression of mosquito populations in large cages as a bridge between lab and field. Nat Commun. 2021;12:4589. 10.1038/s41467-021-24790-6 PMC8319305 34321476

[9] Gantz VM, Jasinskiene N, Tatarenkova O, Fazekas A, Macias VM, Bier E, et al. Highly efficient Cas9-mediated gene drive for population modification of the malaria vector mosquito *Anopheles stephensi*. Proc Natl Acad Sci U S A. 2015;112:E6736-43. 10.1073/pnas.1521077112 PMC4679060 26598698

[10] Pham TB, Phong CH, Bennett JB, Hwang K, Jasinskiene N, Parker K, et al. Experimental population modification of the malaria vector mosquito, *Anopheles stephensi*. PLoS Genet. 2019;15:e1008440. 10.1371/journal.pgen.1008440 PMC6922335 31856182

[11] Hoermann A, Habtewold T, Selvaraj P, Del Corsano G, Capriotti P, Inghilterra MG, et al. Gene drive mosquitoes can aid malaria elimination by retarding sporogonic development. Sci Adv. 2022;8:eabo1733. 10.1126/sciadv.abo1733 PMC9491717 36129981

[12] Eckhoff PA, Wenger EA, Godfray HCJ, Burt A. Impact of mosquito gene drive on malaria elimination in a computational model with explicit spatial and temporal dynamics. Proc Natl Acad Sci U S A. 2017;114:E255-64. 10.1073/pnas.1611064114 PMC5240713 28028208

[13] Hancock PA, North AR, Leach A, Winskill P, Ghani AC, Godfray HCJ, et al. The potential of gene drive releases in malaria vector species to reduce the malaria burden in different African environments. Nat Commun. 2024;15:8976. 10.1038/s41467-024-53065-z PMC11486997 39419965

[14] Mondal A, Sánchez CHM, Marshall JM. A model-informed target product profile for population modification gene drives for malaria control. PLOS Glob Public Health. 2025;5:e0005026. 10.1371/journal.pgph.0005026 PMC12327634 40768510

[15] World Health Organization. How to design vector control efficacy trials: guidance on phase III vector control field trial design provided by the Vector Control Advisory Group. Available from: https://pesquisa.bvsalud.org/portal/resource/pt/who-259688

[16] Marshall JM, North AR. Modeling priorities as gene drive mosquito projects transition from lab to field. In: Mosquito gene drives and the malaria eradication agenda. New York: Jenny Stanford Publishing; 2023. p. 197–237.

[17] Connolly JB, Burt A, Christophides G, Diabate A, Habtewold T, Hancock PA, et al. Considerations for first field trials of low-threshold gene drive for malaria vector control. Malar J. 2024;23:156. 10.1186/s12936-024-04952-9 PMC11110314 38773487

[18] Katusi GC, Hermy MRG, Makayula SM, Ignell R, Govella NJ, Hill SR, et al. Seasonal variation in abundance and blood meal sources of primary and secondary malaria vectors within Kilombero Valley. Southern Tanzania Parasit Vectors. 2022;15:479. 10.1186/s13071-022-05586-z PMC9768911 36539892

[19] Epopa PS, Collins CM, North A, Millogo AA, Benedict MQ, Tripet F, et al. Seasonal malaria vector and transmission dynamics in western Burkina Faso. Malar J. 2019;18:113. 10.1186/s12936-019-2747-5 PMC6444393 30940141

[20] Sougoufara S, Ottih EC, Tripet F. The need for new vector control approaches targeting outdoor biting Anopheline malaria vector communities. Parasit Vectors. 2020;13:295. 10.1186/s13071-020-04170-7 PMC7285743 32522290

[21] Santolamazza F, Mancini E, Simard F, Qi Y, Tu Z, della Torre A. Insertion polymorphisms of SINE200 retrotransposons within speciation islands of *Anopheles gambiae* molecular forms. Malar J. 2008;7:163. 10.1186/1475-2875-7-163 PMC2546427 18724871

[22] Wilkins EE, Howell PI, Benedict MQ. IMP PCR primers detect single nucleotide polymorphisms for *Anopheles gambiae* species identification, Mopti and Savanna rDNA types, and resistance to dieldrin in *Anopheles arabiensis*. Malar J. 2006;5:125. 10.1186/1475-2875-5-125 PMC1769388 17177993

[23] Bass C, Nikou D, Blagborough AM, Vontas J, Sinden RE, Williamson MS, et al. PCR-based detection of Plasmodium in *Anopheles* mosquitoes: a comparison of a new high-throughput assay with existing methods. Malar J. 2008;7:177. 10.1186/1475-2875-7-177. PMC2553798 18793416

[24] Koekemoer LL, Kamau L, Hunt RH, Coetzee M. A cocktail polymerase chain reaction assay to identify members of the *Anopheles funestus* (Diptera: Culicidae) group. Am J Trop Med Hyg. 2002;66:804–11. 10.4269/ajtmh.2002.66.804 12224596

[25] Ji Y, Abrams N, Zhu W, Salinas E, Yu Z, Palmer DC, et al. Identification of the genomic insertion site of Pmel-1 TCR α and β transgenes by next-generation sequencing. PLoS ONE. 2014;9:e96650. 10.1371/journal.pone.0096650 PMC4020793 24827921

[26] Santolamazza F, Caputo B, Calzetta M, Vicente JL, Mancini E, Petrarca V, et al. Comparative analyses reveal discrepancies among results of commonly used methods for *Anopheles gambiae* molecular form identification. Malar J. 2011;10:215. 10.1186/1475-2875-10-215 PMC3170251 21810255

[27] Cierzniak A, Małodobra-Mazur M, Tokarski M. A new approach for the detection of genetic alterations utilizing modified loop-mediated isothermal amplification reaction (LAMP). Sci Rep. 2025;15:8071. 10.1038/s41598-025-93086-2 PMC11889157 40055506

[28] Soroka M, Wasowicz B, Rymaszewska A. Loop-mediated isothermal amplification (LAMP): the better sibling of PCR? Cells. 2021;10:1931. 10.3390/cells10081931 PMC8393631 34440699

[29] Notomi T, Okayama H, Masubuchi H, Yonekawa T, Watanabe K, Amino N, et al. Loop-mediated isothermal amplification of DNA. Nucleic Acids Res. 2000;28:E63. 10.1093/nar/28.12.e63 PMC102748 10871386

[30] Jang WS, Park S, Bae JH, Yoon SY, Lim CS, Cho M-C. Development of a multiplex loop-mediated isothermal amplification (LAMP) for the diagnosis of bacterial periprosthetic joint infection. PLoS ONE. 2024;19:e0302783. 10.1371/journal.pone.0302783 PMC11098349 38753660

[31] Kellner MJ, Koob J, Gootenberg JS, Abudayyeh OO, Zhang F. SHERLOCK: nucleic acid detection with CRISPR nucleases. Nat Protoc. 2019;14:2986–3012. 10.1038/s41596-019-0210-2 PMC6956564 31548639

[32] Bai H, Wang Y, Li X, Guo J. Electrochemical nucleic acid sensors: competent pathways for mobile molecular diagnostics. Biosens Bioelectron. 2023;237:115407. 10.1016/j.bios.2023.115407 37295136

[33] Rašić G, Lobo NF, Jeffrey Gutiérrez EH, Sánchez CHM, Marshall JM. Monitoring Needs for Gene Drive Mosquito Projects: Lessons From Vector Control Field Trials and Invasive Species. Front Genet. 2021;12:780327. 10.3389/fgene.2021.780327 PMC8770328 35069682

[34] Sánchez C HM, Wu SL, Henry JM, Guerra CA, Galick DS, Gárcia GA, et al. Mosquito dispersal in context. PLOS Complex Syst. 2026;3:e0000108.

[35] Huestis DL, Dao A, Diallo M, Sanogo ZL, Samake D, Yaro AS, et al. Windborne long-distance migration of malaria mosquitoes in the Sahel. Nature. 2019;574:404–8. 10.1038/s41586-019-1622-4 PMC11095661 31578527

[36] Carballar-Lejarazú R, Pham TB, Tushar T, James AA. Mutant allele formation and inheritance during Cas9/guide RNA-mediated gene drive in a population modification mosquito strain for human malaria control. Genetics. 2025;231:iyaf176. 10.1093/genetics/iyaf176 PMC12606424 40845150

[37] Werblow A, Flechl E, Klimpel S, Zittra C, Lebl K, Kieser K, et al. Direct PCR of indigenous and invasive mosquito species: a time- and cost-effective technique of mosquito barcoding. Med Vec Entomol. 2015;30:8–13. 10.1111/mve.12154 PMC4982094 26663040

[38] Yigezu A, Zewdie SA, Mirkuzie AH, Abera A, Hailu A, Agachew M, et al. Cost-analysis of COVID-19 sample collection, diagnosis, and contact tracing in low resource setting: The case of Addis Ababa, Ethiopia. PLoS ONE. 2022;17:e0269458. 10.1371/journal.pone.0269458 PMC9182302 35679290

[39] Holst-Jensen A, Bertheau Y, de Loose M, Grohmann L, Hamels S, Hougs L, et al. Detecting un-authorized genetically modified organisms (GMOs) and derived materials. Biotechnol Adv. 2012;30:1318–35. 10.1016/j.biotechadv.2012.01.024 22333321

[40] Broeders SRM, De Keersmaecker SCJ, Roosens NHC. How to deal with the upcoming challenges in GMO detection in food and feed. BioMed Res Int. 2012;2012:402418. 10.1155/2012/402418 PMC3485584 23193359

[41] Wang J, Hu X, Wang Y, Zeng H, Liu X, Liu H. Rapid detection of genetically modified products based on CRISPR-Cas12a combined with recombinase polymerase amplification. Curr Res Food Sci. 2022;5:2281–6. 10.1016/j.crfs.2022.11.009 PMC9691456 36439643

[42] Utarini A, Indriani C, Ahmad RA, Tantowijoyo W, Arguni E, Ansari MR, et al. Efficacy of *Wolbachia*-infected mosquito deployments for the control of dengue. N Engl J Med. 2021;384:2177–86. 10.1056/NEJMoa2030243 PMC8103655 34107180

[43] Indriani C, Tanamas SK, Khasanah U, Ansari MR, Rubangi, Tantowijoyo W, et al. Impact of randomised *w*mel *Wolbachia* deployments on notified dengue cases and insecticide fogging for dengue control in Yogyakarta City. Glob. Health Action. 2023;16:2166650. 10.1080/16549716.2023.2166650 PMC9894080 36700745

